# Growth-induced percolation on complex networks

**DOI:** 10.1093/pnasnexus/pgaf192

**Published:** 2025-06-11

**Authors:** Hongliang Sun, Shuhuan Chen, Jiarong Xie, Yanqing Hu

**Affiliations:** College of Wealth Management, Ningbo University of Finance and Economics, No.899 Xueyuan Road, Haishu District, Ningbo, Zhejiang 315175, China; Institute of Data Space, Hefei Comprehensive National Science Center, No.288 Innovation Avenue, Gaoxin District, Hefei, Anhui 231283, China; Department of Statistics and Data Science, College of Science, Southern University of Science and Technology, No.1088 Xueyuan Avenue, Nanshan District, Shenzhen, Guangdong 518055, China; Center for Computational Communication Research, Beijing Normal University, No.18, Jinfeng Road, Xiangzhou District, Zhuhai, Guangdong 519087, China; School of Journalism and Communication, Beijing Normal University, No.19, Xinjiekouwai St, Haidian District, Beijing 100875, China; Department of Statistics and Data Science, College of Science, Southern University of Science and Technology, No.1088 Xueyuan Avenue, Nanshan District, Shenzhen, Guangdong 518055, China; Center for Complex Flows and Soft Matter Research, Southern University of Science and Technology, No.1088 Xueyuan Avenue, Nanshan District, Shenzhen, Guangdong 518055, China

**Keywords:** complex networks, percolation, phase transition, social networks, indirect influences

## Abstract

Empirical studies have increasingly highlighted the crucial role of indirect social interactions in shaping human behaviors, yet theoretical models have largely focused on direct influences. By analyzing scientific collaboration networks, we demonstrate that direct and indirect collaborators are key in triggering high-impact research periods. Inspired by these findings, we propose a novel model, growth-induced percolation, which captures how individuals are activated through indirect interactions. Our model reveals a striking asymmetry in the hysteresis loop between growth-induced percolation and its reverse process, with distinct phase transition behaviors. Our work provides a foundational framework for understanding how indirect interactions drive the spread of behaviors in social systems, with implications for fields ranging from scientific collaboration to social contagion.

Significance Statement There is growing empirical evidence from a variety of systems that indirect interactions play a key role in shaping new behaviors. We propose growth-induced percolation, the first model to demonstrate how individuals are activated through indirect interaction. Our model is supported by empirical data showing that the quality of a scientist’s publications is influenced by his/her two-degree collaborators. We find that the hysteresis loop is asymmetric in the phase transition type between growth-induced percolation and its reversal process. This indirect mechanism spreads new behavior with a very different outcome than the direct impact.

## Introduction

Percolation is one of the classical models of statistical physics, providing illustrative highlights with phase transition and critical behavior (1, 2). Although the rules are very simple, percolation theory is widely used to describe many real-world systems (3–5). Early studies of percolation focused on regular lattices (1), which are widely used in conducting materials (6) and magnetic materials (7). With the rapid development of network science (8, 9), the applications of percolation theory has increased steadily in recent years (5). Considering the interaction between pairs of nodes, site percolation and bond percolation in the networks are used to study the cascading of infrastructure failures (10–13), the spread of diseases (14), and the information propagation (15, 16) in social networks (17). In addition to the two-body interactions, network-specific percolation models are proposed to study multibody interactions, such as linear threshold percolation (18), bootstrap percolation (19), *k*-core percolation (20, 21) and its generalization (22), core percolation (23), optimal percolation (24), explosive percolation (25), percolation in interdependent networks (26–28), and hyper networks (29).

The above studies mainly focus on the direct interactions between the nearest neighbors. To study the indirect interactions in many real-world systems (30–32), Xie et al. (33) proposed the retention-induced percolation, which shows that second-order collaborators play a dominant role in the retention of scientists in old research fields. However, indirect interactions also play an important role in the spreading dynamics of emergent behaviors and affective states. It is discovered that behaviors like obesity and affective states like happiness, alcohol, and drugs are influenced by indirect social contagion with underlying social connections (34). In this article, we propose the growth-induced percolation to describe the growth of a small number of active nodes activate others through indirect influences. The term *growth* specifically distinguishes the node activation (inactive-to-active) process from the inactivation process in previous retention-induced percolation (33). The activation process leads to a different type of phase transition from the retention-induced percolation and the proliferation model with addition or division of nodes (35).

In particular, there is a symmetry of the giant component size in directed and undirected Erdös–Rényi (ER) random networks. Furthermore, the hysteresis loop is asymmetric between growth-induced percolation and retention-induced percolation in undirected ER networks. We found that continuous and hybrid phase transitions occur in ER networks. While in scale-free (SF) networks, hub nodes can trigger avalanche first-order phase transitions. It concludes that the underlying degree distribution of the network dominates the order of the phase transition. Furthermore, the growth-induced effect is supported by the empirical data on publication quality, both indirect and direct collaborators play a significant role in the hot streaks (high performance periods in career) of scientists in the collaboration network.

## Results

**Fig. 1.**
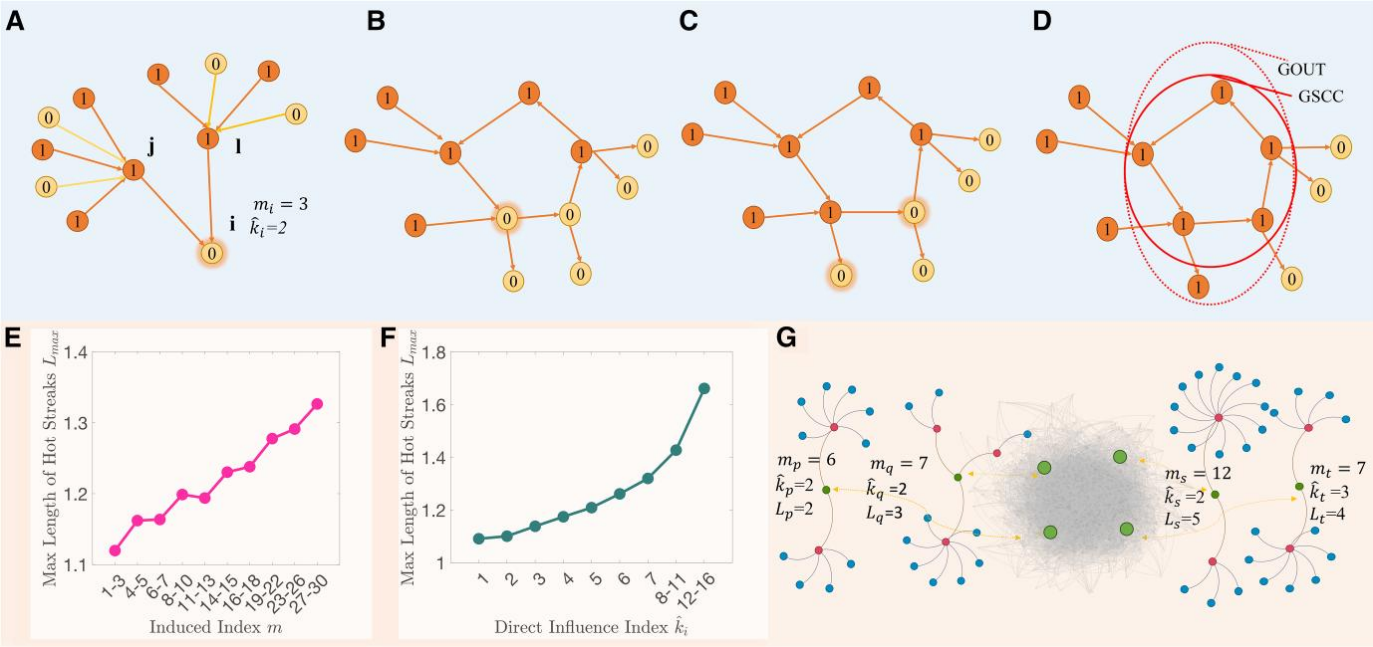
Illustration of the dynamic propagation of growth-induced percolation, in which a small number of initially active nodes trigger state transitions in others through second-neighbor effects. A) Definition of induce index. Node *i* contains two active incoming neighbors *j* and *l*, node *j* has three active incoming neighbors and node *l* contains 2. The maximum of these two values is the induced index $m_{i}=\max\{2,3\}=3$. B–D) The evolution of a toy network with 12 nodes under the dynamic propagation of growth-induced percolation with threshold m=2. B) The initial state of the toy directed network with six initially active nodes. The highlighted node will be activated because its induced index $m_{i}=2$ is not less than the threshold m=2. C) The evolution of the dynamic. Two more nodes will be activated. D) The final state and giant component of the model. The giant strongly connected component (GSCC) with five nodes in the solid circle and the giant out-component (GOUT) with 6 nodes in the dashed circle. E, F) The maximal length of hot streaks $L_{\max}$ as a function of the indirect and the direct influence index, respectively. The co-author network contains 560,396 computer scientists (nodes) and 2,073,703 edges between them. Each index range contains about 10% of scientist who did not have a hot streak in 2002–2005. G) Visualization of the indexes in the co-author network. Node p,q,s have the same direct scores, while node *s* has maximum value of $m_{s}$, which corresponds to the maximum value of $L_{s}$. Similar results can be obtained from node *s* and node *t*, since their direct influences are quite close and indirect impact determines the length of hot streak *L*. See SI Section S1, S2 and Figs. S1, S2 for more details.

We propose the growth-induced percolation, which shows how a small number of active nodes can trigger other nodes to become active via indirect influences. Specifically, each node is initially active with probability *q* and inactive with probability 1−q. We label active nodes as state 1 and inactive nodes as state 0. A node *i* with state 0 will change to 1 if its induced index $m_{i}$ is not less than the given threshold *m* (i.e. $m_{i}\geq m$). In other words, if at least one of *i*’s incoming neighbors *j* is active, and node *j* contains at least *m* active incoming neighbors, *j* will activate *i* to state 1. Otherwise, node *i* remains state 0. This process is repeated until no node changes its state. For example, Fig. 1A shows $m_{i}=3$ because the induced index $m_{i}$ denotes the maximum number of active second-nearest neighbors. Figure 1B–D demonstrates the dynamic process of a toy network. Empirical study on high performance periods of scientists from DBLP has been carried out from Fig. 1E–G. It concludes that in addition to direct impacts, indirect collaborations have also contributed to the spreading of hot streaks of scientists.

We use mean-field theory to solve the model in directed networks with arbitrary degree distributions. The equations in this section and those in Methods section provide exact solutions for tree-like networks. Given a randomly chosen link i→j, we define *x* as the probability that the node *i* is in state 1. And *y* as the probability that node *i* can induce node *j* to state 1 (see Table 1). For *x*, if *i* is initialized as 1, it will remain state 1. If node *i* is initialized as 0, then it needs at least one incoming neighbors to induce it active. The probability of such a process is $1-(1-y{)}^{k_{\mathrm{in}}}$, where $k_{\mathrm{in}}$ is the in-degree of *i*. The degree distribution of node *i* is $\frac{k_{\mathrm{out}}P(k_{\mathrm{in}},k_{\mathrm{out}})}{\left\langle k\right\rangle }$, where $k_{\mathrm{out}}$, $P(k_{\mathrm{in}},k_{\mathrm{out}})$ and ⟨k⟩ denote the out-degree of *i*, the degree distribution of the network, and average degree of the network, respectively. Thus, the self-consistent equation of *x* is:


(1) $$x=q+(1-q)\sum_{k_{\mathrm{in}},k_{\mathrm{out}}}\frac{k_{\mathrm{out}}P(k_{\mathrm{in}},k_{\mathrm{out}})}{\left\langle k\right\rangle }\left[1-(1-y{)}^{k_{\mathrm{in}}}\right].$$

**Table 1.** Definition of probabilities for the theoretical solution of growth-induced percolation in directed networks.

Notation	Occurring event
*x*	*i* is active
*y*	*i* can induce *j*
$x_{\infty }$	*j* connects to GOUT through node *i*, given *j* is active
$y_{\infty }$	*i* can induce *j*, and *j* connects to GOUT through node *i*

The first column shows the notation used to denote the probability that the event in the second column occurs for a randomly chosen directed link i→j.

For *y*, if *i* is initialized as 1, the event *i* can induce *j* requires that the number of *i*’s incoming neighbors *s* is s≥m, the probability is (kins)xs(1−x)kin−s. If the initial state is 0, except s≥m, it is also required that at least one of the *s* incoming neighbors can induce *j*, the probability is $1-(1-\frac{y}{x}{)}^{s}$. Thus, the self-consistent equation of *y* is:


(2) y=q∑kin,koutkoutP(kin,kout)⟨k⟩∑s=mkin(kins)xs(1−x)kin−s+(1−q)∑kin,koutkoutP(kin,kout)⟨k⟩*∑s=mkin(kins)xs(1−x)kin−s[1−(1−yx)s].

$P_{a}$ denotes the proportion of final active node:


(3) $$P_{a}=q+(1-q)\sum_{k_{\mathrm{in}},k_{\mathrm{out}}}P(k_{\mathrm{in}},k_{\mathrm{out}})\left[1-(1-y{)}^{k_{\mathrm{in}}}\right].$$

The deduction of $P_{a}$ is similar to *x*, and it is omitted here.

Next, we calculate the GOUT size $P_{\infty }$. For a randomly chosen link i→j, we define $x_{\infty }$ as the probability that *j* connects to GOUT through node *i*, given the condition that *j* is active. And we define $y_{\infty }$ as the probability that both two events are true: *i* can induce *j*, and *j* connects to GOUT through node *i*. See SI Section S3 for the definition of GOUT.

For $x_{\infty }$, if *i* is initialized as state 1 (with probability *q*, see the former term of right-hand size of Eq. 5), the event *j* connecting to GOUT through node *i* requires that *i* connects to GOUT through at least one of *i*’s incoming neighbors. The probability is $1-(1-x_{\infty }{)}^{k_{\mathrm{in}}}$. If *i* is initialized as state 0 (with probability 1−q, see the latter term of right-hand size of Eq. 5), it requires t>0 incoming neighbors to induce *i*, the probability is (kint)yt(1−y)kin−t. *i* may connect to GOUT through the $k_{\mathrm{in}}$ incoming neighbors. For one of the *t* neighbors *l*, the probability that *i* connects to GOUT through *l* is $\frac{y_{\infty }}{y}$, given the condition that *l* can induce *i*. For one of the $k_{\mathrm{in}}-t$ neighbors *l*, the probability that *i* connects to GOUT through *l* is $\frac{x_{\infty }-y_{\infty }}{1-y}$, given the condition that *l* cannot induce *i*. Therefore, the probability that *i* connects to GOUT is:


(4) ∑t=1kin(kint)yt(1−y)kin−t[1−(1−y∞y)t(1−x∞−y∞1−y)kin−t]=1−(1−y)kin−(1−x∞)kin+(1−y−x∞+y∞)kin.

For simplicity, the right side of Eq. 4 is denoted by $H(y,x_{\infty },y_{\infty };k_{\mathrm{in}})=1-(1-y{)}^{k_{\mathrm{in}}}-(1-x_{\infty }{)}^{k_{\mathrm{in}}}+(1-y-x_{\infty }+y_{\infty }{)}^{k_{\mathrm{in}}}$. $H(y,x_{\infty },y_{\infty };k_{\mathrm{in}})$ denotes the probability for a randomly chosen link i→j that *j* connects to GOUT through node *i*, given the following two conditions: (i) *i* is initialized as state 0 and (ii) the degree of *i* is $\left(k_{\mathrm{in}},k_{\mathrm{out}}\right)$. The self-consistent equation of $x_{\infty }$ is:


(5) $$\begin{array}{rl} x_{\infty } & =q\sum_{k_{\mathrm{in}},k_{\mathrm{out}}}\frac{k_{\mathrm{out}}P(k_{\mathrm{in}},k_{\mathrm{out}})}{\left\langle k\right\rangle }\left[1-(1-x_{\infty }{)}^{k_{\mathrm{in}}}\right]\\ & \;+(1-q)\sum_{k_{\mathrm{in}},k_{\mathrm{out}}}\frac{k_{\mathrm{out}}P(k_{\mathrm{in}},k_{\mathrm{out}})}{\left\langle k\right\rangle }H(y,x_{\infty },y_{\infty };k_{\mathrm{in}}). \end{array}$$

For $y_{\infty }$, the event *i* can induce *j* requires that in *i*’s $k_{\mathrm{in}}$ incoming neighbors, s≥m neighbors are active, the probability is (kins)xs(1−x)kin−s. If *i* is initialized as 1, the event *j* connects to GOUT through node *i* requires that *i* connects to GOUT through at least one of its *s* active incoming neighbors. The probability is $1-(1-\frac{x_{\infty }}{x}{)}^{s}$. If *i* is initialized as 0, in the *s* active incoming neighbors, the event requires t>0 incoming neighbors to induce *i*, the probability is (st)(yx)s(1−yx)s−t. *i* may connect to GOUT through the *s* incoming neighbors. For one of the *t* neighbors *l*, the probability that *i* connects to GOUT through *l* is $\frac{y_{\infty }}{y}$, given the condition that *l* can induce *i*. For one of the s−t neighbors *l*, the probability that *i* connects to GOUT through *l* is $\frac{x_{\infty }-y_{\infty }}{x-y}$, given the condition that *l* is active but cannot induce *i*. Therefore, the probability that *i* connects to GOUT is:


(6) ∑t=1s(st)(yx)s(1−yx)s−t*[1−(1−y∞y)t(1−x∞−y∞x−y)s−t]=1−(1−yx)s−(1−x∞x)s+(1−y+x∞−y∞x)s=H(yx,x∞x,y∞x;s).

The self-consistent equation of $y_{\infty }$ is:


(7) y∞=q∑kin,koutkoutP(kin,kout)⟨k⟩*∑s=mkin(kins)xs(1−x)kin−s[1−(1−x∞x)s]+(1−q)∑kin,koutkoutP(kin,kout)⟨k⟩*∑s=mkin(kins)xs(1−x)kin−sH(yx,x∞x,y∞x;s).

Finally, the deduction of the probability $P_{\infty }$ that a randomly chosen node *i* belongs to GOUT is similar to the deduction of $x_{\infty }$, except that the degree distribution of *i* is $P(k_{\mathrm{in}},k_{\mathrm{out}})$ rather than $\frac{k_{\mathrm{out}}P(k_{\mathrm{in}},k_{\mathrm{out}})}{\left\langle k\right\rangle }$. Therefore,


(8) $$\begin{array}{rl} P_{\infty } & =q\sum_{k_{\mathrm{in}},k_{\mathrm{out}}}P(k_{\mathrm{in}},k_{\mathrm{out}})\left[1-(1-x_{\infty }{)}^{k_{\mathrm{in}}}\right]\\ & \;+(1-q)\sum_{k_{\mathrm{in}},k_{\mathrm{out}}}P(k_{\mathrm{in}},k_{\mathrm{out}})H(y,x_{\infty },y_{\infty };k_{\mathrm{in}}). \end{array}$$

**Fig. 2.**
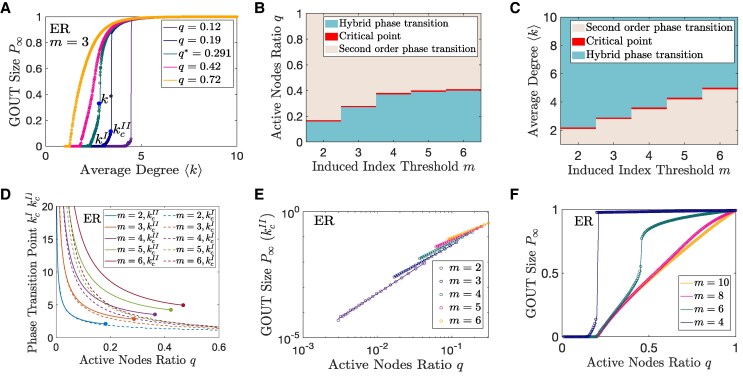
Phase transitions of growth-induced percolation in directed ER networks. A) The relation between the GOUT size $P_{\infty }$ and the average degree ⟨k⟩. The solid blue dots indicate the critical point $k^{*}$, the phase transition point $k_{c}^{I}$, and the jumping point $k_{c}^{II}$. B) The types of phase transition in the m−q plane. C) The types of phase transition in the m−⟨k⟩ plane. D) The relation between the phase transition points $k_{c}^{I}$, $k_{c}^{II}$ and the active node ratio *q*. The solid dots indicate the critical points between continuous transitions and hybrid transitions. E) The relation between the GOUT size $P_{\infty }$ at the jump point $k_{c}^{II}$ and the active node ratio *q*. Circles represent numerical solutions and straight lines represent linear fits in log–log coordinates. F) The relations between GOUT size $P_{\infty }$ and *q* with different *m*. In A and F), hollow dots represent simulation results and curves represent theoretical results.

We first study the phase transition and critical behavior in ER networks in Fig. 2. The simplified theoretical solution in directed ER networks can be found in SI Section S4. Figures 2A and S3 show that when the initial active ratio *q* is large, there is a continuous phase transition of $P_{\infty }$ from zero to nonzero at $k_{c}^{I}$. On the other hand, when *q* is small, the system exhibits a hybrid phase transition. Specifically, $P_{\infty }$ changes continuously at $k_{c}^{I}$ and jumps steeply at $k_{c}^{II}$. The boundary between continuous transition and hybrid transition is the critical point $q^{*}$. The jump height approaches 0 as *q* approaches $q^{*}$ at the right-hand side. The phase transition diagram is shown in Fig. 2B and C. More results of growth-induced percolation in directed ER networks are shown in SI Section S5, Figs. S3 and S4.

Next, we show the absent of first-order phase transition on directed ER networks. Figure 2D shows that $k_{c}^{I}$ and $k_{c}^{II}$ decreases rapidly with *q* when *q* is small. Figure 2E shows that $P_{\infty }(k_{c}^{II})$ increases as a power function of *q*, indicating that $k_{c}^{I}$ and $k_{c}^{II}$ do not overlap, and it concludes that there is no first-order phase transition in directed ER networks.

**Fig. 3.**
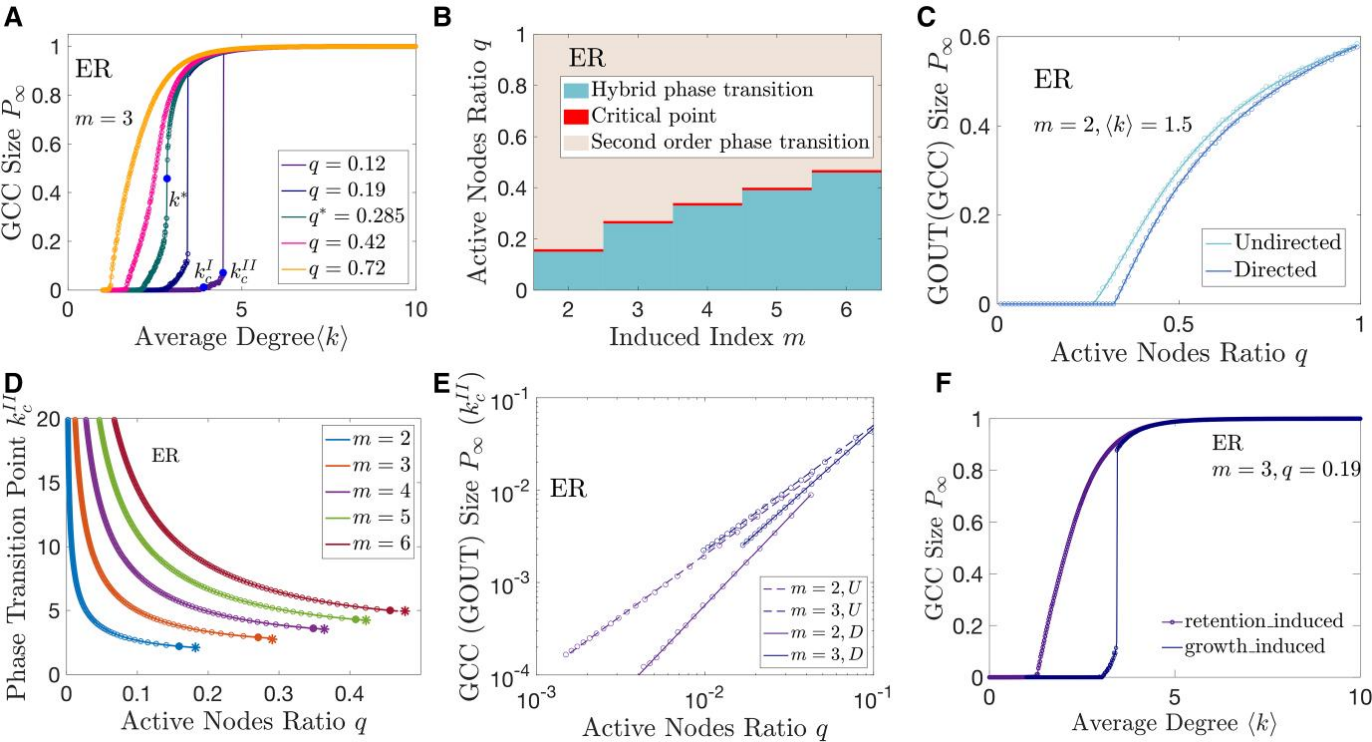
Phase transitions of growth-induced percolation in undirected ER networks. A) The relations between GCC size $P_{\infty }$ and average degree ⟨k⟩ in ER networks. The solid dot indicate the critical point $k^{*}$, the continuous phase transition point $k_{c}^{I}$, and the jump point $k_{c}^{II}$. B) The types of phase transition in m−q plane with different *m*. C) Comparison of GCC size $P_{\infty }$ on undirected ER networks and GOUT size $P_{\infty }$ on directed ER networks. ⟨k⟩=1.5,m=2. D) The relation between the jump points $k_{c}^{II}$ and active node ratio *q*, where circles indicate undirected ER networks and lines denote directed ER networks. Solid dots at the end of circle curves denote the critical points between continuous and hybrid phase transitions of undirected ER networks. Asterisks denote the critical points of directed ER networks. E) The relations between GCC size or GOUT size at the jump point $k_{c}^{II}$ and active node ratio *q*. Circles represent numerical solutions and straight lines represent linear fits in log–log coordinates. F) The asymmetric of hysteresis loop of growth-induced percolation and retention-induced percolation. Growth-induced percolation shows hybrid phase transition and retention-induced percolation displays continuous phase transition. m=3,q=0.19.

The mechanism and theoretical solution of growth-induced percolation in undirected networks is shown in Methods section, SI Section S6, Fig. S5, and Table S2. Figure 3 shows that the phase transition behavior of growth-induced percolation is very similar between directed and undirected networks. In particular, the ratio of active nodes in directed and undirected ER networks is exactly the same (see SI Section S4), but both $P_{\infty }$ and $k_{c}^{I}$ are slightly different (see Fig. 3C), and the jumping point $k_{c}^{II}$ is very close (see Figs. 3D and S6). More results of growth-induced percolation in directed ER networks are shown in Figs. S7–S9.

It is shown in Fig. 3F that the hysteresis loop is asymmetric in phase transition type between growth-induced percolation and retention-induced percolation. To be specific, there is hybrid phase transitions in growth-induced percolation and continuous phase transitions in retention-induced percolation. For comparability, we fixed *q* proportion of nodes to state 1 in retention-induced percolation, the rest of nodes may change to state 0 by the retention-induced mechanism. This is because *q* proportion of nodes are initially active. This model is equivalent to a heterogeneous retention-induced percolation mode in which *q* proportion of nodes with threshold 0 and 1−q proportion of nodes with threshold *m* (36). Note that the steady state of growth-induced percolation is also the steady state of heterogeneous retention-induced percolation, and vice versa.

**Fig. 4.**
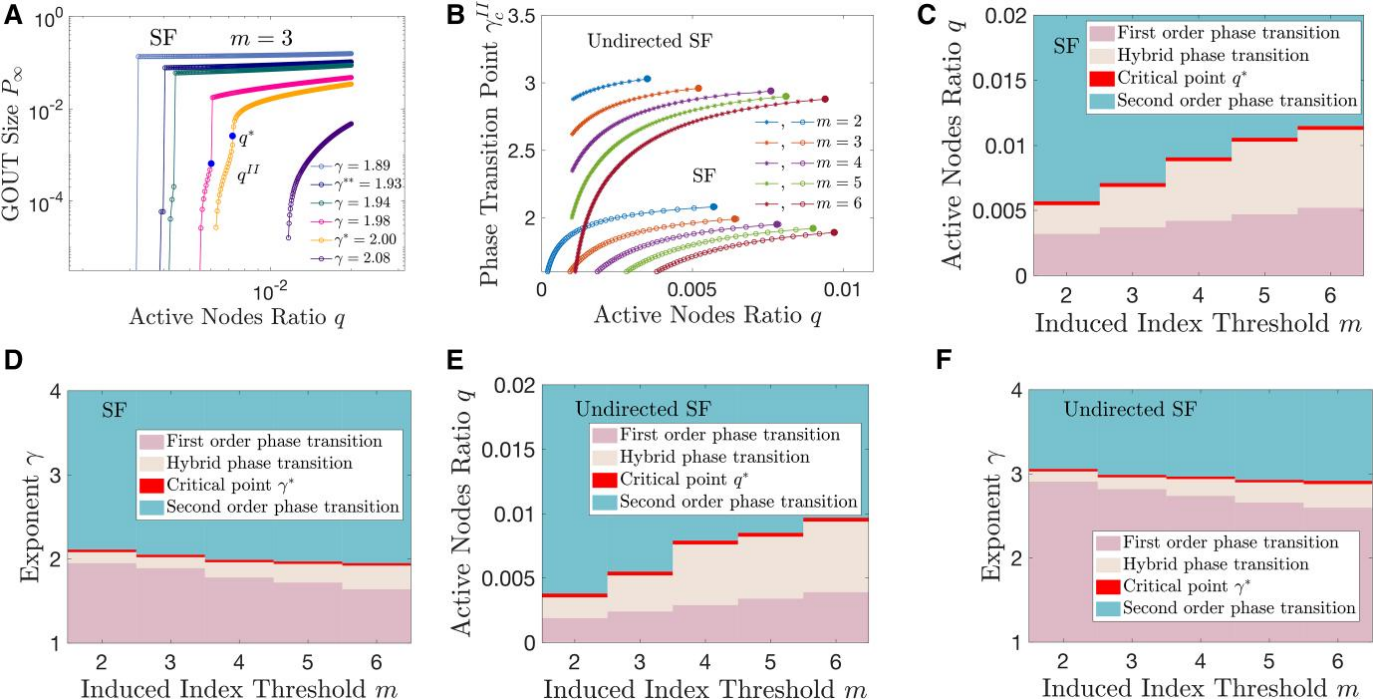
Phase transition of growth-induced percolation in directed and undirected SF networks. A) The relation between the GOUT size $P_{\infty }$ and active node ratio *q* in directed SF networks. The solid dots indicate the critical point $q^{*}$, and the jump point $q^{II}$. B) The relation between the jump point $\gamma_{c}^{II}$ and active node ratio *q* in directed and undirected SF networks. Solid dots at the end of the curves indicate critical points of hybrid and second-order phase transitions. C–F) The types of phase transition in directed and undirected SF networks. In directed SF networks, $1\leq k_{\mathrm{in}},k_{\mathrm{out}}\leq 100$ without degree correlation, i.e. $P(k_{\mathrm{in}},k_{\mathrm{out}})\propto k_{\mathrm{in}}^{-\gamma }*k_{\mathrm{out}}^{-\gamma }$. In undirected SF networks, 1≤k≤100 and $P(k)\propto k^{-\gamma }$.

The phase transition of growth-induced percolation in SF networks are different from that in ER networks. Apart from the second-order and hybrid phase transition, there is a first-order phase transition in SF networks (see Fig. 4). The examination of phase transition order is shown in SI Section S7 and Fig. S10. More results of growth-induced percolation in SF networks are shown in SI Section S8 and Figs. S11–S15. The comparison of the phase transition behavior of growth-induced percolation between directed and undirected networks is very different from that of retention-induced percolation (33) (see Table 2). In particular, the directionality of the links in retention-induced percolation determines the type of phase transition. In growth-induced percolation, however, the degree distribution determines the type of phase transition, while the directionality does not affect it.

**Table 2.** Comparison of the phase transition types between growth-induced percolation and induced percolation.

Network type	Growth-induced percolation			Retention-induced percolation		
	Continuous	Hybrid	First-order	Continuous	Hybrid	First-order
Directed ER	√	√	×	√	×	√
Directed SF	√	√	√	√	×	√
Undirected ER	√	√	×	√	×	×
Undirected SF	√	√	√	√	×	×

√ indicates the phase transition type present in the networks. × indicates the phase transition type not present in the networks.

**Fig. 5.**
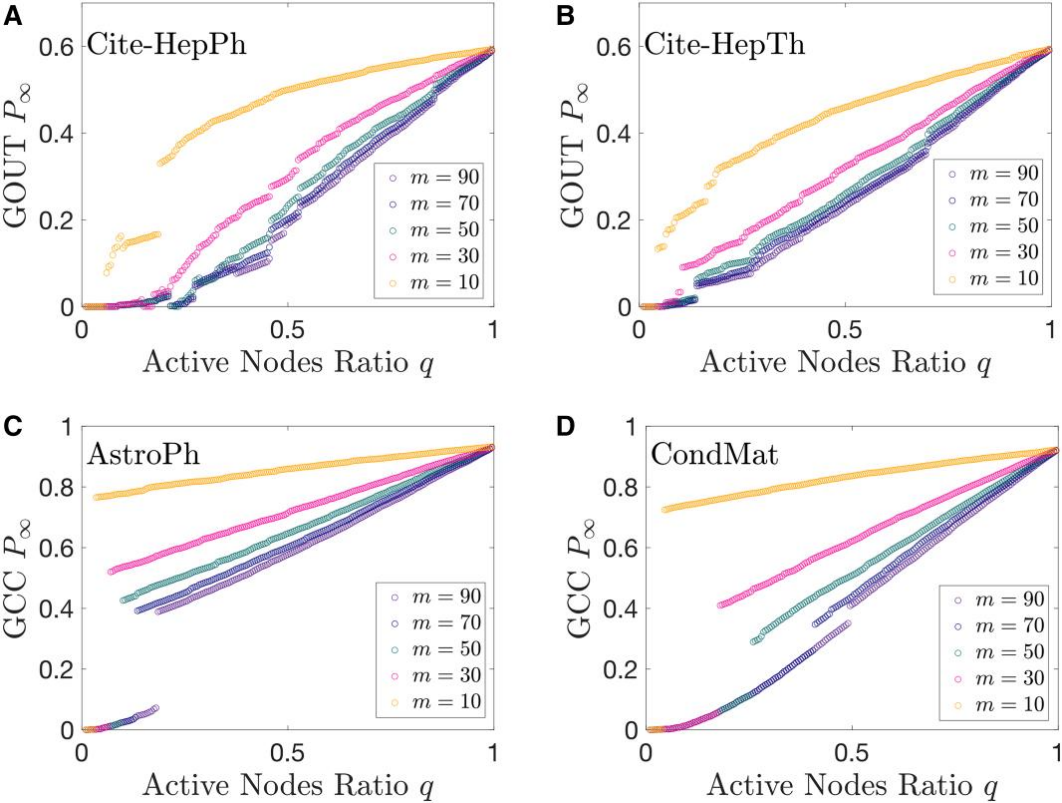
Growth-induced percolation in real-world scholar collaboration networks. See Table S1 for descriptions of the networks.

The results in real-world collaboration networks show the similar phase transition behaviors to artificial networks. Figure 5A–D show that there are continuous phase transitions when *m* is large and hybrid phase transition when *m* is small. This phenomenon is consistent in line with artificial networks (see Fig. 2F).

## Conclusion

Retention-induced percolation (33) is a percolation model to study how indirect influences can affect the spread of social behavior, such as scientist collaboration, happiness, or social emotions in social systems. It assumes that all participants are initially active, with some becoming inactive if they do not meet the inducing effects. We propose a new model, growth-induced percolation, which aims to uncover the reverse process by demonstrating how a set of active nodes can trigger other inactive nodes to become active. By examining this mechanism in collaboration data among scientists, we find that both indirect and direct neighbors induce hot streaks in scientists.

Growth-induced percolation exhibits a completely different phase transition phenomenon compared to retention-induced percolation. In retention-induced percolation, the directionality of the links determines the phase transition types, with a first-order phase transition occurring when the ratio of directed links is high. In contrast, degree distributions play a crucial role in the phase transitions in growth-induced percolation. We observe both continuous and hybrid phase transitions in directed and undirected ER networks. While there is a small gap in the giant component size between directed and undirected ER networks, the final ratio of active nodes is the same. Furthermore, it is important to discover the hysteresis loop is asymmetric between growth-induced percolation and retention-induced percolation in undirected ER networks. Further studies on real-world networks reveal hybrid, and continuous phase transitions, consistent with theoretical studies in artificial networks.

Compared to retention-induced percolation, this novel model illustrates the growth mechanism of the spreading process via indirect effects, including phase transitions and critical behaviors. It can be applied to investigate the dynamics of spreading behaviors in social systems driven by a small number of active individuals, such as social contagion of behaviors and emotions. In future work, we plan to investigate the implications of growth-induced percolation in multilayer networks, which could provide deeper insights into the role of indirect interactions across interconnected systems (28).

## Methods

### Theoretical solution in undirected networks

The mechanism of growth-induced percolation in undirected networks are shown in SI Section S6. To theoretically analyze the GCC size $P_{\infty }$, we start by defining five conditional probabilities as intermediate variables, whose notations are shown in Table S2. Without loss of generality, we denote a randomly chosen undirected link as i−j and deduce the probability that node *i* belongs to a GCC. The degree distribution of node *j* in the chosen link is $\frac{kP(k)}{\left\langle k\right\rangle }$, where P(k) is the degree distribution of the network.

We define $\tilde{x}$ as the probability that the node *j* is in state 1 without the induction from node *i*. And $\tilde{y}$ as the probability that *j* can induce node *i* to state 1. For $\tilde{x}$, with probability *q*, *j* is initialized as 1 and will not change. Otherwise with probability 1−q, *j* is initialized as 0, *j* requires at least one of its k−1 neighbors (excluding *i*) to induce it, the probability is $1-(1-\tilde{y}{)}^{k-1}$. Therefore, the self-consistent equation of $\tilde{x}$ is:


(9) $$\tilde{x}=q+(1-q)\sum_{k}\frac{kP(k)}{\left\langle k\right\rangle }\left[1-(1-\tilde{y}{)}^{k-1}\right].$$

For $\tilde{y}$, if the initial state of *j* is 1, the event *j* can induce *i* requires that the number of *j*’s incoming neighbors *s* is s≥m. The probability is F(x~;k−1,s)=(k−1s)x~s(1−x~)k−1−s. In case of no confusion, it is abbreviated as $F(s)=F(\tilde{x};k-1,s)$. If *j*’s initial state is 0, except s≥m, it also requires that at least one of the *s* neighbors can induce *j*, the probability is $1-(1-\frac{\tilde{y}}{\tilde{x}}{)}^{s}$. Therefore, the self-consistent equation of $\tilde{y}$ is:


(10) $$\begin{array}{rl} \tilde{y} & =q\sum_{k}\frac{kP(k)}{\left\langle k\right\rangle }\sum_{s=m}^{k-1}F(s)+(1-q)\\ & \;*\sum_{k}\frac{kP(k)}{\left\langle k\right\rangle }\sum_{s=m}^{k-1}F(s)\left[1-{\left(1-\frac{\tilde{y}}{\tilde{x}}\right)}^{s}\right]. \end{array}$$

${\hat{P}}_{a}$ denotes the proportion of active nodes:


(11) $${\hat{P}}_{a}=q+(1-q)\sum_{k}P(k)\left[1-(1-\tilde{y}{)}^{k}\right].$$

The deduction of ${\hat{P}}_{a}$ is similar to $\hat{x}$, and it is omitted here.

Next, we calculate the GCC size $P_{\infty }$. For a randomly chosen link i−j, we define *α* as the probability that node *i* connect to GCC through node *j*, under the condition that node *i* can induce node *j* to state 1. We define *β* as the probability that both the event corresponding to $\tilde{x}$ and *α* occur and define *γ* as the probability that both the event corresponding to $\tilde{y}$ and *β* occur. In notation, β=Pr(X∧A), γ=Pr(Y∧B), in which X,Y,A,B denotes the event corresponding to the probability $\tilde{x},\tilde{y},\alpha ,\beta$, respectively.

For *α*, given *i* can induce *j* to state 1, the initial state of *j* has no effect. If the number of active neighbors of *j* (excluding *i*) satisfies s≤m−2, *j* cannot induce its remaining k−1−s inactive neighbors. In this case, *α* requires that *j* connect to GCC through the *s* neighbors. For one of the *s* neighbors *l*, the probability that *j* connects to GCC through *l* is $\frac{\beta }{\tilde{x}}$ given the condition that *l* is active. Therefore, the probability that *i* connects to GCC through *j* is $1-(1-\frac{\beta }{\tilde{x}}{)}^{s}$. If s≥m−1, *j* can induce its remaining k−1−s neighbors to state 1. Except for the *s* neighbors, *j* may connect to GCC through the k−1−s neighbors. For one of the k−1−s neighbors *l*, the probability that *j* connects to GCC through *l* is $\frac{\alpha -\beta }{1-\tilde{x}}$, given the condition that *l* is state 0 without the induction of *j*. Therefore, the probability that *i* connects to GCC through *j* is $1-(1-\frac{\beta }{\tilde{x}}{)}^{s}(1-\frac{\alpha -\beta }{1-\tilde{x}}{)}^{k-1-s}$. The self-consistent equation of *α* is:


(12) $$\begin{array}{rl} \alpha & =\sum_{k}\frac{kP(k)}{\left\langle k\right\rangle }\sum_{s=0}^{m-2}F(s)\left[1-{\left(1-\frac{\beta }{\tilde{x}}\right)}^{s}\right]+\sum_{k}\frac{kP(k)}{\left\langle k\right\rangle }\\ & \;*\sum_{s=m-1}^{k-1}F(s)\left[1-{\left(1-\frac{\beta }{\tilde{x}}\right)}^{s}{\left(1-\frac{\alpha -\beta }{1-\tilde{x}}\right)}^{k-1-s}\right]. \end{array}$$

For β=Pr(X∧A), if the initial state of node *j* is 1, it is no different from *α*. If the initial state is 0, due to the event *X*, at least one of *j*’s neighbors can induce *j*. In *j*’s *s* active neighbors (without the induction from *j*), *t* neighbors can induce *j*, the probability is F(y~x~;s,t)=(st)(y~x~)t(1−y~x~)s−t (we abbreviate it as G(t)), and the event *X* requires that t>0. If s≤m−2, the event *A* requires that *j* connects to GCC through the *s* neighbors. For one of the *t* neighbors *l*, the probability that *j* connects to GCC through *l* is $\frac{\gamma }{\tilde{y}}$ given the condition that *l* can induce *j*. For one of the s−t neighbors *l*, the probability that *j* connects to GCC through *l* is $\frac{\beta -\gamma }{\tilde{x}-\tilde{y}}$ given the condition that *l* is active but cannot induce *j*. Therefore, the probability is:


(13) ∑t=1s(st)(y~x~)t(1−y~x~)s−t[1−(1−γy~)t(1−β−γx~−y~)s−t]=1−(1−y~x~)s−(1−βx~)s+(x~−y~−β+γx~)s.

If s≥m−1, *j* can induce its remaining k−1−s neighbors to state 1. Except for the *s* neighbors, *j* may connect to GCC through the k−1−s neighbors. Similar to the deduction of *α*, the probability that *j* connects to GCC through one of its k−1−s neighbors is $\frac{\alpha -\beta }{1-\tilde{x}}$. Therefore, the probability is:


(14) ∑t=1s(st)(y~x~)t(1−y~x~)s−t*[1−(1−γy~)t(1−β−γx~−y~)s−t(1−α−β1−x~)k−1−s]=1−(1−y~x~)s−(1−βx~)s(1−α−β1−x~)k−1−s+(x~−y~−β+γx~)s(1−α−β1−x~)k−1−s.

The self-consistent equation of *β* is:


(15) β=qα+(1−q)∑kkP(k)⟨k⟩∑s=0m−2F(s)*[1−(1−y~x~)s−(1−βx~)s+(x~−y~−β+γx~)s]+(1−q)∑kkP(k)⟨k⟩∑s=m−1k−1F(s)[1−(1−y~x~)s(y~x~)k−s−(1−βx~)s(1−α−β1−x~)k−1−s+(x~−y~−β+γx~)s(1−α−β1−x~)k−1−s].

For γ=Pr(Y∧B), the event *Y* requires that *j* has s≥m active neighbors (excluding *i*). The event *Γ* is a subset of the event *B*, and *Γ* consists of the case of s≥m in event *B*. Therefore, the self-consistent equation of *γ* is


(16) γ=q∑kkP(k)⟨k⟩∑s=mk−1F(s)[1−(1−βx~)s(1−α−β1−x~)k−1−s].+(1−q)∑kkP(k)⟨k⟩∑s=mk−1F(s)[1−(1−y~x~)s(y~x~)k−s−(1−βx~)s(1−α−β1−x~)k−1−s+(x~−y~−β+γx~)s(1−α−β1−x~)k−1−s].

Finally, the deduction of the probability $P_{\infty }$ that a randomly chosen node *j* belongs to GCC is similar to the deduction of *β*, except that the degree distribution of *j* is P(k) rather than $\frac{kP(k)}{\left\langle k\right\rangle }$, and the condition that *j* can induce its k−s neighbors is s≥m rather than s≥m−1. Therefore,


(17) P∞=q∑kP(k)∑s=0m−1F(s)[1−(1−βx~)s]+q∑kP(k)∑s=mkF(s)[1−(1−βx~)s(1−α−β1−x~)k−s]+(1−q)∑kP(k)∑s=0m−1F(s)*[1−(1−y~x~)s−(1−βx~)s+(x~−y~−β+γx~)s]+(1−q)∑kP(k)∑s=mkF(s)[1−(1−y~x~)s(y~x~)k−s−(1−βx~)s(1−α−β1−x~)k−s+(x~−y~−β+γx~)s(1−α−β1−x~)k−s].

## Supplementary Material

pgaf192_Supplementary_Data

## Data Availability

The datasets were derived from sources in the public domain: Symposium on Database Programming Languages (DBPL), https://dblp.org/db/conf/dbpl/index.html, and Stanford Network Analysis Project (SNAP), http://snap.stanford.edu/data/. The code for this study is available at https://github.com/hlsun84/growth-induced-percolation.
