# Supplementary material for: Growth-induced percolation on complex networks
Source: PNAS Nexus. 2025 Jun 11;4(6):pgaf192. doi: 10.1093/pnasnexus/pgaf192 (PMC12199759; doi:10.1093/pnasnexus/pgaf192)
Supplement: pgaf192_Supplementary_Data [file pgaf192_supplementary_data.pdf]

PNAS nexus

Supplementary Material for

Hongliang Sun, Shuhuan Chen, Jiarong Xie and Yanqing Hu

Jiarong Xie
E-mail: jrxie@bnu.edu.cn

This PDF file includes:

Supplementary text
Figs. S1 to S15
Tables S1 to S2

Supplementary Material Text

S1. Real-world networks data set

We present more details about the empirical study of real-world networks here. Five empirical networks are involved to investigate how growth-induced percolation mechanisms are behaved. DBLP co-authorship network is applied to investigate the impacts of direct influences and indirect influences on how hot streaks of computer scientists evolve. This data set is collected from the year 2002 to the year 2011. Ccollaboration networks (AstroPh, CondMat) anc citation networks (Cite-HepPh,Cite-HepTh) are analyzed to understand the indirect influences of one individual's behavior or actions on others in the network, even without direct interactions. AstroPh collaboration network is from the e-print arXiv and covers scientific collaborations between authors papers submitted to Astro Physics category. If an author i co-authored a paper with author j, the graph contains a undirected edge from i to j. CondMat collaboration network covers scientific collaborations between authors papers submitted to Condense Matter category. Cite-HepPh covers all the citations from Arxiv high energy physics phenomenology. If a paper i cites paper j, the graph contains a directed edge from i to j. Cite-HepTh network is a paper citation network of Arxiv High Energy Physics category. Details about topological properties are shown in Table. S1.

S2. Inducing effects in empirical data

Details of the analysis of the DBLP dataset are presented here. The hot streak highlights a specific period during which an individual's performance is significantly better than his/her typical performance. We empirically study how the hot streaks are influenced by the state of the neighborhood.

A. Definitions and notations. Using this DBLP data, we construct a collaboration network. Specifically, a node in the network represents a scientist who has at least one publication from 2002 to 2011 in the dataset. An edge between two nodes represents that the two scientists are co-authors on at least one publication from 2002 to 2011 in the dataset.

In addition to the network structure, we also define the state of scientists. For each publication, we count the number of citations until 2022. We define highly cited publications of a scientist as those exceed his/her median citation. Note that a highly cited publication of one author may not be a highly cited publication of another author. We define a period of consecutive highly cited publications as a hot streak period of a scientist, and define the number of publications during the hot streak period as the length L, which is illustrated in Fig. S1. If a highly cited publication is sandwiched between two lowly cited publications, we still define $L = 1$.

To study the change in the state of the scientist, we divide the ten-year period into two phases: the first four years (2002 to 2005) and the last six years (2006 to 2011). We define state 0 as a scientist who has no hot streak period in the first four years, and state 1 as a scientist who has at least one hot streak period in the first four years. Combining the state of scientists and the collaboration network, we measure the direct influence index $\hat{k}_i$, which is the number of its nearest state 1 neighbors, and the induced index m_i of scientist i.

B. Supplementary empirical results. We study the correlation between the length of hot streak periods in the last six years and the indexes in the first four years for those state 0 scientists. Two-way ANOVA (Analysis of Variance) is a statistical technique used to analyze the impact of two different categorical independent factors on a continuous dependent variable. It assesses the main effects of each factor as well as the interaction effect between the factors. Fig. S2 illustrates a positive correlation between the maximal length of hot streaks and both m_i and $\hat{k}_i$. The p-value obtained by two-way ANOVA is $p(m) = 9.63 * 10^{-57}, p(\hat{k}_i) = 0, p(m, \hat{k}_i) = 1.06 * 10^{-381}$, which means that both direct and indirect neighbors have a significant effect on the state of scientists. These results imply that the significant effect of direct and indirect neighbors on the state of scientists is robust.

S3. Order parameters in growth-induced percolation

We investigated two order parameters, giant connected component (GCC) and giant out-component (GOUT). GCC is used in undirected network where it denotes the largest connected component. GOUT is used in directed networks where it denotes the group of nodes that can be reached from any node in giant strongly connected component (GSCC). Specifically, all active nodes form a set of strongly connected components, the largest of which is the GSCC. During the theoretical study of ER, SF and empirical networks, we use P_∞ to demonstrate the GSCC in undirected networks and GOUT in directed networks.

S4. Simplified theoretical solution of growth-induced percolation in ER networks

In the main text, we give the theoretical solution of growth-induced percolation with arbitrary degree distributions. By substituting the Poisson distribution of ER networks, the solutions are simplified. Such a simplified formula can be used to efficiently speed up the numerical solutions.

A. In directed ER networks. Here, we show the theoretical solution of growth-induced percolation in directed ER networks. The degree distribution is a Poisson distribution in directed ER random networks is:

$$P(k_{\mathrm{in}}) = \frac{c^{k_{\mathrm{in}}} e^{-c}}{k_{\mathrm{in}}!}, \quad [1]$$

$$P(k_{\text{out}}) = \frac{c^{k_{\text{out}}} e^{-c}}{k_{\text{out}}!}, \tag{2}$$

$$P(k_{\text{in}}, k_{\text{out}}) = P(k_{\text{in}}) P(k_{\text{out}}), \tag{3}$$

where c is the average degree.

The self-consistent equations of x and y are simplified as:

$$x = 1 - (1-q) e^{-cy}, \tag{4}$$

$$y = x - \sum_{k_{\text{in}}} P(k_{\text{in}}) \left\{ \sum_{s=0}^{\min\{m-1, k_{\text{in}}\}} \binom{k_{\text{in}}}{s} \left[x^s - (1-q)(x-y)^s\right] (1-x)^{k_{\text{in}}-s} \right\}, \tag{5}$$

The final active node ratio is:

$$P_a = 1 - (1-q) e^{-cy} = x. \tag{6}$$

The self-consistent equations of x_∞ and y_∞ are simplified as:

$$x_\infty = 1 - e^{-cx_\infty} - (1-q) e^{-cy} + (1-q) e^{-c(y + x_\infty - y_\infty)}, \tag{7}$$

$$\begin{aligned} y_\infty = x_\infty - \sum_{k_{\text{in}}} P(k_{\text{in}}) \Bigg\{ & \sum_{s=0}^{\min\{m-1, k_{\text{in}}\}} \binom{k_{\text{in}}}{s} (1-x)^{k_{\text{in}}-s} \\ & * \left[x^s - (x - x_\infty)^s - (1-q)(x-y)^s + (1-q)(x - y - x_\infty + y_\infty)^s\right] \Bigg\}. \end{aligned} \tag{8}$$

Finally, P_∞ is simplified as:

$$P_\infty = 1 - e^{-cx_\infty} - (1-q) e^{-cy} + (1-q) e^{-c(y - x_\infty + y_\infty)} = x_\infty. \tag{9}$$

B. In undirected ER networks. Here, we show the theoretical solution of growth-induced percolation in undirected ER networks. The degree distribution is a Poisson distribution in undirected ER random networks is:

$$P(k) = \frac{c^k e^{-c}}{k!}, \tag{10}$$

where c is the average degree.

The self-consistent equations of $\tilde{x}$, $\tilde{y}$ and $\tilde{P}_a$ are simplified as:

$$\tilde{x} = 1 - (1-q) e^{-c\tilde{y}}, \tag{11}$$

$$\begin{aligned} \tilde{y} &= 1 - (1-q) e^{-c\tilde{y}} \\ &\quad - \sum_k \frac{kP(k)}{c} \left\{ \sum_{s=0}^{\min\{m-1, k\}} \binom{k-1}{s} (1-\tilde{x})^{k-1-s} \left[\tilde{x}^s - q(\tilde{x} - \tilde{y})^s\right] \right\} \\ &= \tilde{x} - \sum_k Q(k) \left\{ \sum_{s=0}^{\min\{m-1, k\}} \binom{k}{s} (1-\tilde{x})^{k-s} \left[\tilde{x}^s - (1-q)(\tilde{x} - \tilde{y})^s\right] \right\}, \end{aligned} \tag{12}$$

where $Q(k) = \frac{(k+1)P(k+1)}{c}$. In ER networks $Q(k) = P(k)$. The final active node ratio is:

$$\hat{P}_a = 1 - (1-q) e^{-c\tilde{y}} = \tilde{x}. \tag{13}$$

The self-consistent equations of α, β and γ are simplified as:

$$\begin{aligned} \alpha &= 1 - e^{-c\alpha} \\ &\quad - \sum_k Q(k) \left\{ \sum_{s=0}^{\min\{m-2, k\}} \binom{k}{s} (\tilde{x} - \beta)^s \left[(1-\tilde{x})^{k-s} - (1 - \tilde{x} - \alpha + \beta)^{k-s}\right] \right\}, \end{aligned} \tag{14}$$

$$\beta = q\alpha + (1-q)\left[1 - e^{-c\tilde{y}} - e^{-c\alpha} + e^{-c(\tilde{y}+\alpha-\gamma)}\right]$$
$$- (1-q)\sum_k Q(k)\left\{\sum_{s=0}^{\min\{m-2,k\}} \binom{k}{s} \left[(\tilde{x}-\beta)^s - (\tilde{x}-\tilde{y}-\beta+\gamma)^s\right]\right. \quad [15]$$
$$\left. * \left[(1-\tilde{x})^{k-s} - (1-\tilde{x}-\alpha+\beta)^{k-s}\right]\right\},$$

$$\gamma = 1 - e^{-c\alpha} - (1-q)e^{-c\tilde{y}} + (1-q)e^{-c(\tilde{y}+\alpha-\gamma)}$$
$$- \sum_k Q(k)\left\{\sum_{s=0}^{\min\{m-1,k\}} \binom{k}{s} \left\{\tilde{x}^s(1-\tilde{x})^{k-s} - (\tilde{x}-\beta)^s(1-\tilde{x}-\alpha+\beta)^{k-s}\right.\right. \quad [16]$$
$$\left.\left. -(1-q)\left[(\tilde{x}-\tilde{y})^s(1-\tilde{x})^{k-s} - (\tilde{x}-\tilde{y}-\beta+\gamma)^s(1-\tilde{x}-\alpha+\beta)^{k-s}\right]\right\}\right\}.$$

Finally, P_∞ is simplified as:

$$P_\infty = 1 - e^{-c\alpha} - (1-q)e^{-c\tilde{y}} + (1-q)e^{-c(\tilde{y}+\alpha-\gamma)}$$
$$- \sum_k P(k)\left\{\sum_{s=0}^{\min\{m-1,k\}} \binom{k}{s} \left[(\tilde{x}-\beta)^s - (1-q)(\tilde{x}-\tilde{y}-\beta+\gamma)^s\right]\right. \quad [17]$$
$$\left. * \left[(1-\tilde{x})^{k-s} - (1-\tilde{x}-\alpha+\beta)^{k-s}\right]\right\}.$$

C. Equal final active node ratio between directed and undirected ER networks. One can check that x, y, P_a in directed ER networks are exactly the same as $\hat{x}$, $\hat{y}$, and $\hat{P}_a$ in undirected ER networks, by substituting the degree distribution $Q(k) = \frac{(k+1)P(k+1)}{c} = P(k)$ in undirected ER networks.

S5. Supplementary results in ER networks

Figs. S3 and S4 show more results of growth-induced percolation in directed ER networks. It is known that there are hybrid phase transitions with small q value and continuous phase transitions with large q values in Fig. S3. Similarly, there are hybrid phase transitions with large $\langle k\rangle$ value and continuous phase transitions with small $\langle k\rangle$ values in Fig. S4. The phase transition types are mainly shown in details in Fig. S7. To be specific, we have interests to explore how phase transition points k_c^I change with respect to active node ratio q in Fig. S6. It is shown that difference of k_c^I in terms of q on directed and on directed ER networks are small, which are demonstrated by close curves with same parameter settings. Such characters keep in line with the gap of GOUT (GCC) in terms of q in main context.

And Figs. S7, S8, S9 show more results of growth-induced percolation in undirected ER networks. There are the same phase transition types and similar trends compared with the case in directed ER networks. The trends of these figures are the same with Figs. 2 and 3 in the main text.

S6. Mechanism of growth-induced percolation in undirected networks

The mechanism of growth-induced percolation in undirected networks is similar to that in directed networks. A node i with state 0 can be changed to 1 if its induced index m_i is not less than the given threshold m ($m_i \geq m$). In other words, if at least one of i's neighbors j is active, and node j contains at least m active neighbors, j will activate i to state 1. Otherwise, node i remains state 0. This process is repeated until no node changes its state, as illustrated in Fig. S5.

S7. Examination of phase transition order

The order can be determined by whether there is a peak of second largest connected component (the strongly connected component in directed networks) and of the number of iterative iterations (NOI). When the system exhibits continuous phase transition, there is a peak of SC and no peak of NOI (see Fig. S10 B, D). When the system exhibits hybrid phase transition, there are non-overlapping peak of SC at phase transition point, and peak of NOI at the jumping point (see Fig. S10 A, C, F, H). When the system exhibits first-order phase transition, there is a peak of NOI and no peak of SC (see Fig. S10 E, G).

S8. Supplementary results in SF networks

We show more results of growth-induced percolation in SF networks. The degree distribution of scale-free networks is $P(k) \propto k^{-\gamma}$ in the range $k_{\min} \leq k \leq k_{\max}$. If without a special request, $k_{\min} = 1$ and $k_{\max} = 1000$ in this paper.

Figs. S11 and S12 show more results in directed SF networks, and Figs. S13 and S14 show more results in undirected SF networks. Fig. S15 shows that the theoretical results are in good agreement with the simulation results. In the simulation, the networks are constructed using configuration model with degree distribution of scale-free networks is $P(k) \propto k^{-\gamma}$ in the range $k_{\min} \leq k \leq k_{\max}$, $k_{\min} = 1$ and $k_{\max} = 100$.

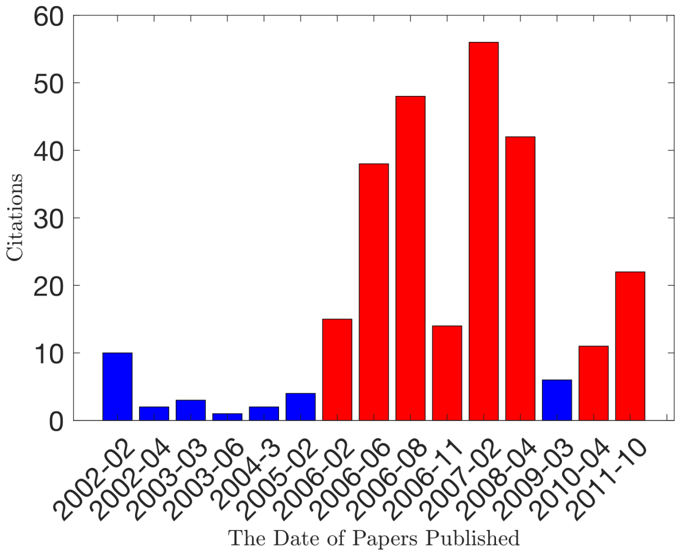

Fig. S1. An illustration of the hot streaks of a scientist who published 15 papers between 2002 and 2011. The x-axis is the publication month and the y-axis is the number of citations of the paper. The median number of citations for 15 papers is 11. A hot streak is a series of consecutive papers with citations above the median. There are two hot streaks with lengths $L = 6$ (2006-02 to 2008-04) and $L = 2$ (2010-04 to 2011-10).

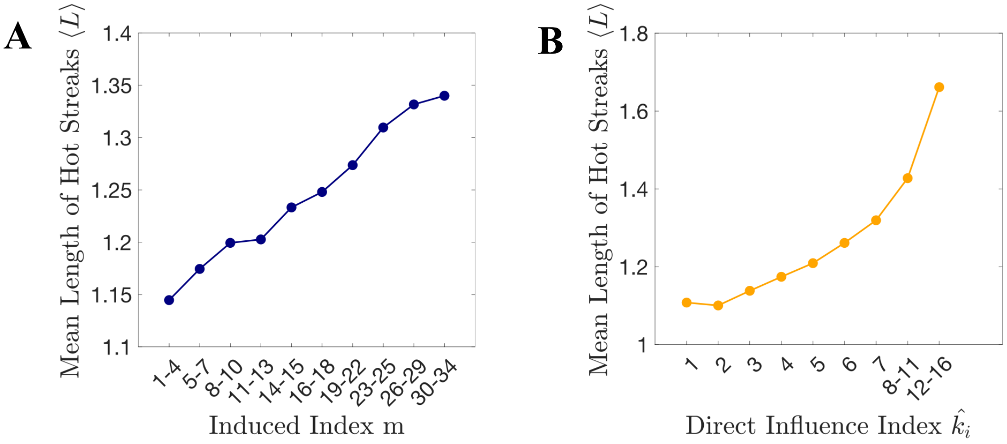

Fig. S2. The mean length of hot streaks $\langle L \rangle$ as a function of the direct and indirect influence. Each index range contains about 10% of scientist who did not have a hot streak in 2002-2005.

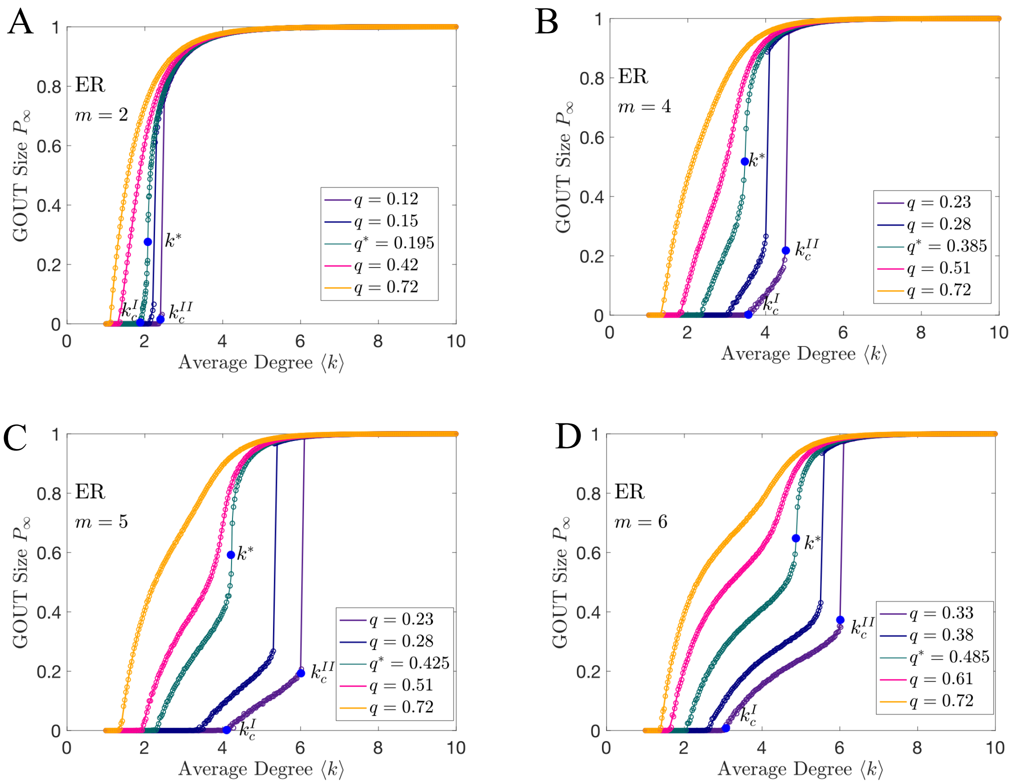

Fig. S3. The relation between GOUT size P_∞ and the average degree $\langle k \rangle$ in directed ER networks. There are continuous phase transitions with large q value and hybrid phase transitions with small q value. The critical points q^* and k^* indicate the boundary between such two phase transitions. In all figures, the blue solid dots indicate the critical point k^*, the phase transition point k_c^I, and the jumping point k_c^{II}. Curves indicate theoretical solutions and dots represent simulations in large networks with network size 1,000,000.

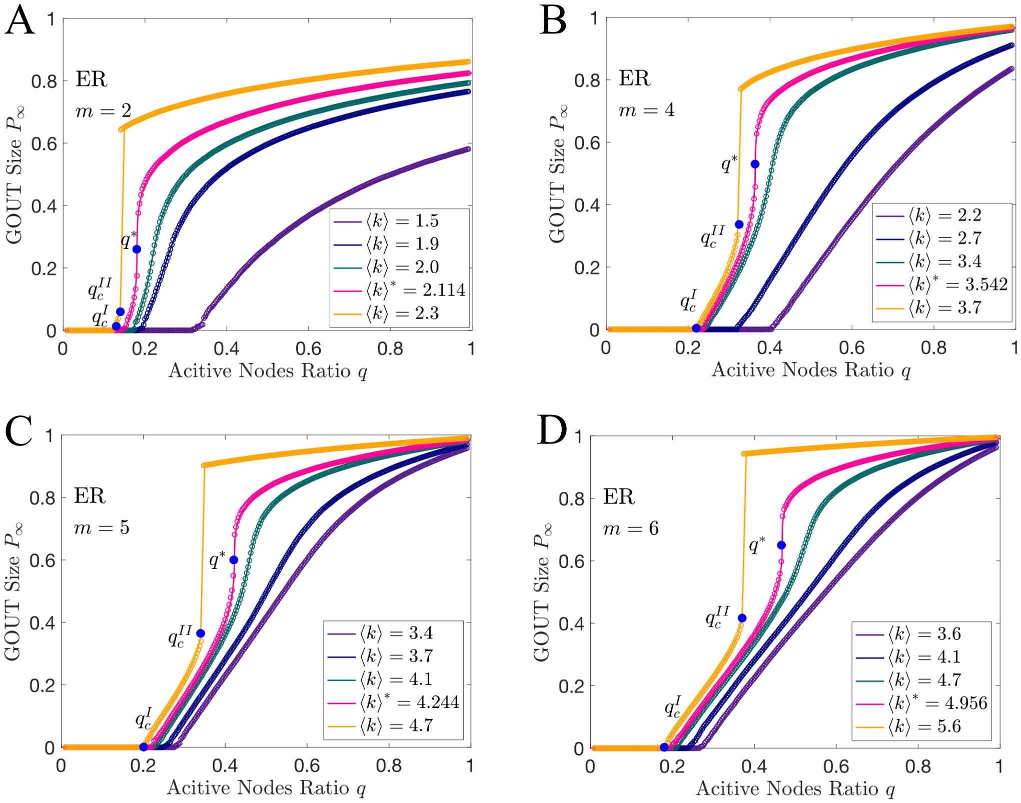

Fig. S4. The relation between GOUT size P_∞ and the active node ratio q in directed ER networks. There are continuous phase transitions with small $\langle k \rangle$ value and hybrid phase transitions with large $\langle k \rangle$ value. The critical points q^* and k^* indicate the boundary between such two phase transitions. In all figures, the blue solid dots indicate the critical point q^*, the phase transition point q_c^I, and the jumping point q_c^{II}. Curves indicate theoretical solutions and dots represent simulations in large networks with network size 1,000,000.

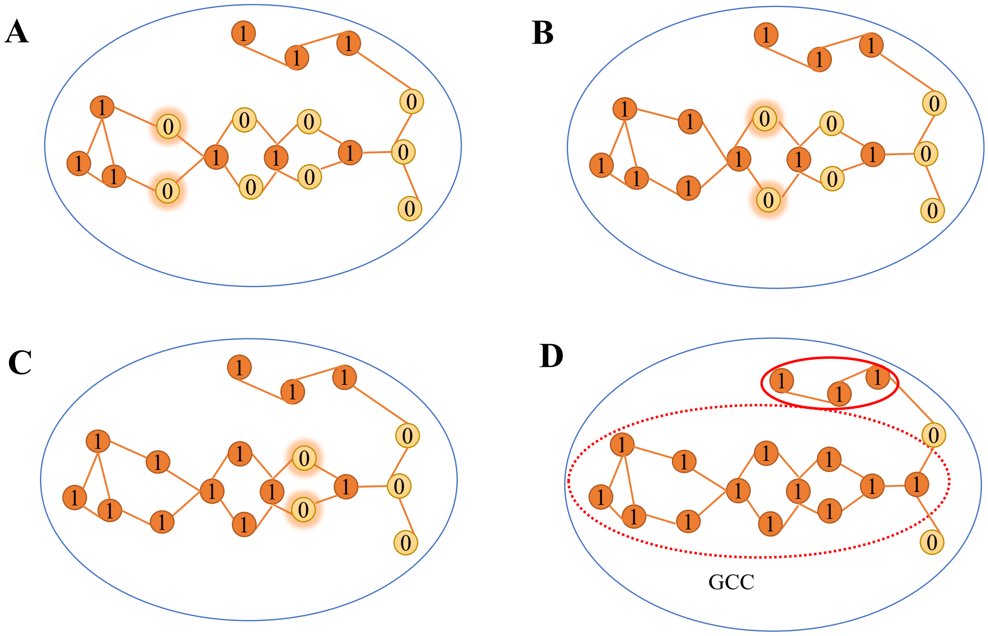

Fig. S5. Illustration of growth-induced percolation dynamic in undirected networks. The induced index $m = 2$, meaning that if node j has at least 2 active neighbors (excluding i), j will activate its neighbor i. (**A**) The initial state where 9 out of 18 nodes are active. (**B**), (**C**) Evolution of growth-induced percolation dynamic in undirected networks. (**D**) The final state of the network. The activate nodes construct two connect components and the largest one is the giant connected component (GCC).

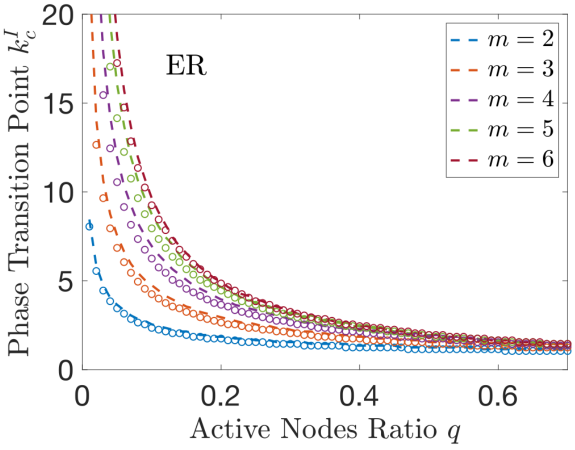

Fig. S6. The relation between the phase transition points k_c^I and active node ratio q in directed and undirected ER networks. Circles represent k_c^I with respect of q in directed ER networks. Dashed lines denote k_c^I with respect of q in undirected ER networks. It concludes that the differences of k_c^I with respect of q on directed and undirected ER networks are very small.

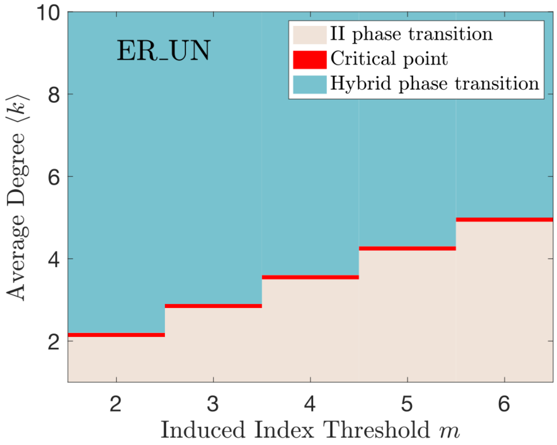

Fig. S7. The types of phase transition in $m - \langle k \rangle$ plane in undirected ER networks. There are continuous phase transitions and hybrid phase transitions separated by solid red lines as critical points. The inducing index value m increases as positive integer and the critical point increases as well.

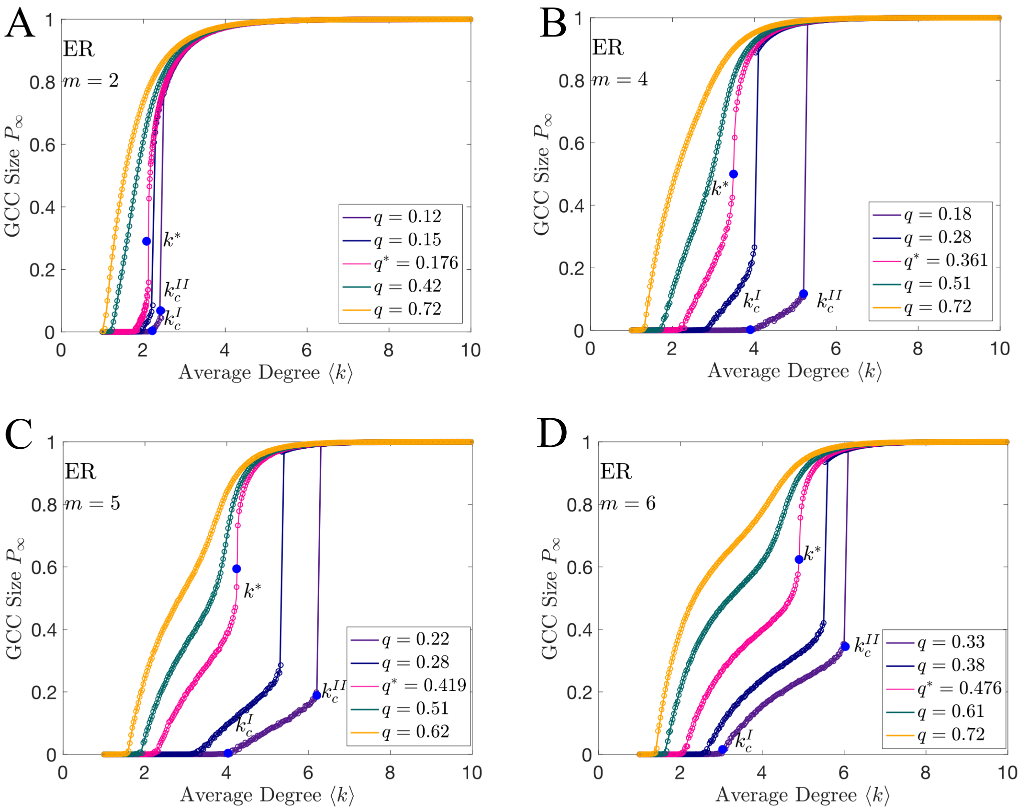

Fig. S8. The relation between GCC size P_∞ and the average degree $\langle k \rangle$ in undirected ER networks. There are continuous phase transitions with large q value and hybrid phase transitions with small q value. The boundary between such two phase transitions are critical points q^* and k^*. In all figures, the blue solid dots indicate the critical point k^*, the phase transition point k_c^I, and the jumping point k_c^{II}. Curves indicate theoretical solutions and dots represent simulations in large networks with network size 1,000,000.

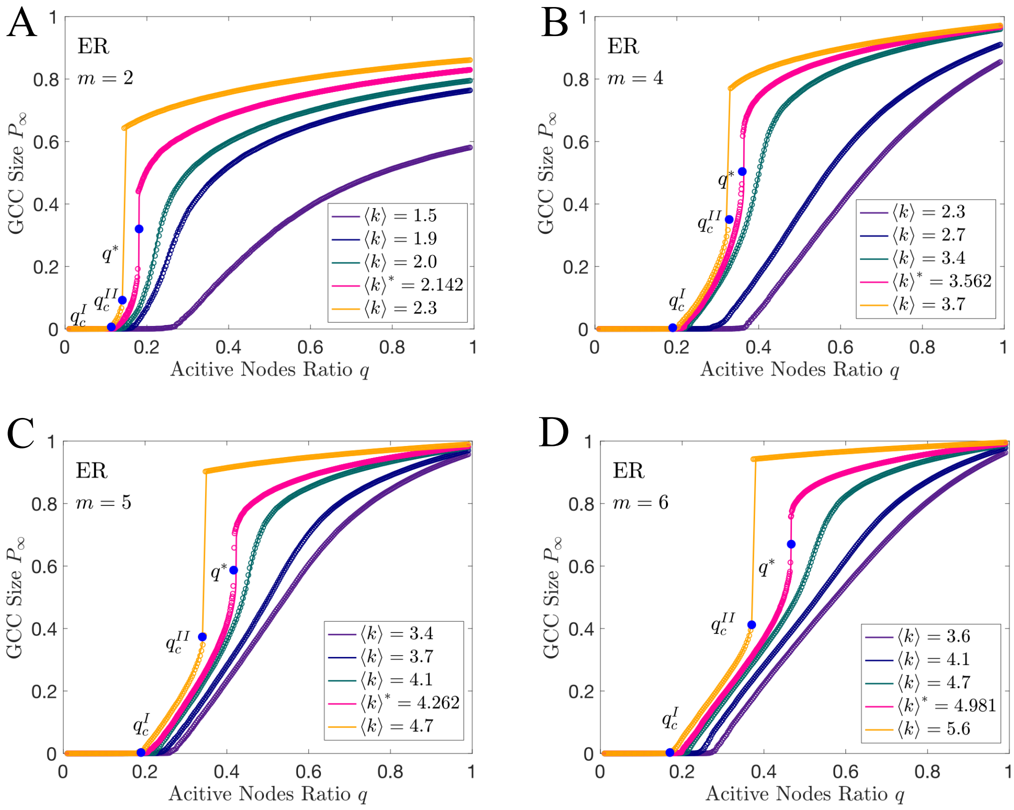

Fig. S9. The relation between GCC size P_∞ and active mode ratio q in undirected ER networks. There are continuous phase transitions with small $\langle k \rangle$ value and hybrid phase transitions with large $\langle k \rangle$ value. The boundary between such two phase transitions are critical points q^* and k^*. In all figures, the blue solid dots indicate the critical point q^*, the phase transition point q_c^I, and the jumping point q_c^{II}. Curves indicate theoretical solutions and dots represent simulations in large networks with network size 1,000,000.

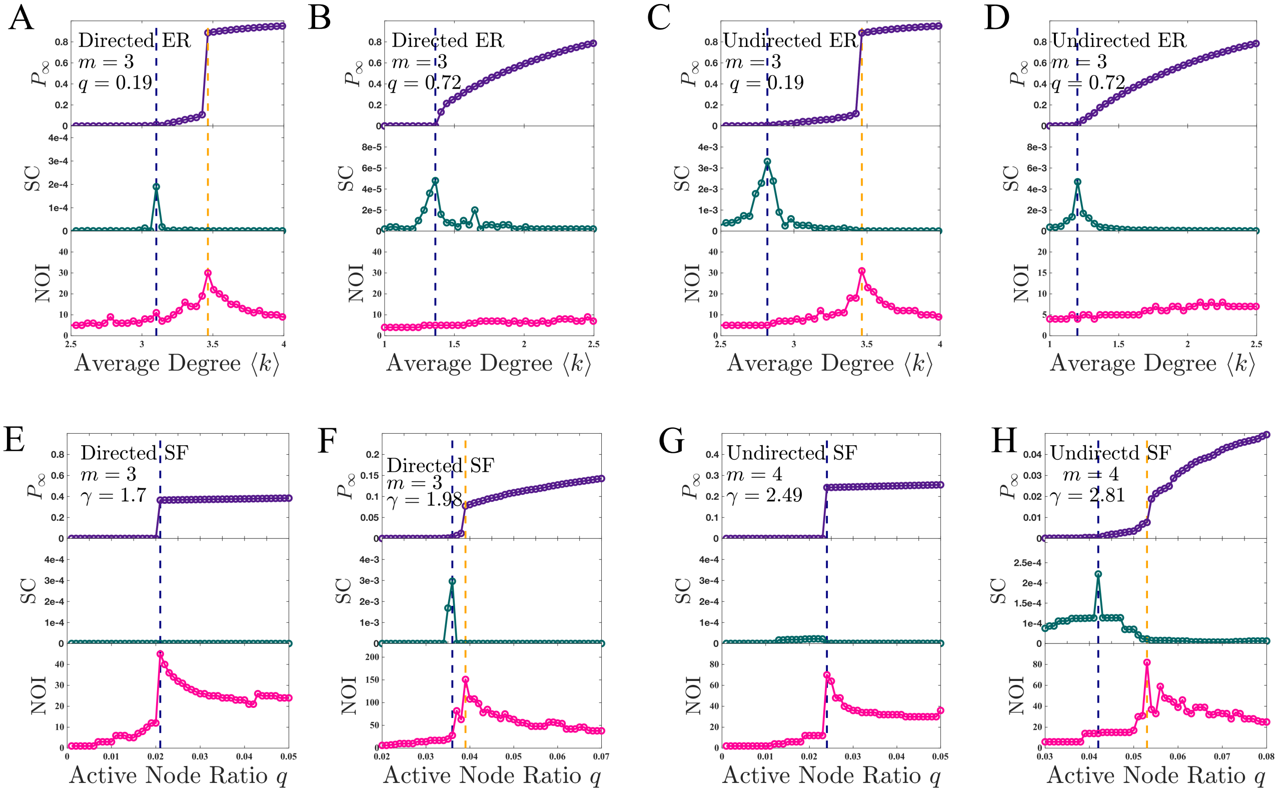

Fig. S10. Examination of phase transition order of growth-induced percolation on different networks. Within each subfigure, from top to bottom is: Top: The GCC size for undirected networks or the GOUT size for directed networks. Middle: The size of the second largest connected component in undirected networks or the size of second largest strongly connected component in directed networks. Bottom: The number of iterative iterations (NOI). The network size is 1,000,000 in subfigures (**A**)-(**D**) and 5,000,000 in subfigures (**E**)-(**H**).

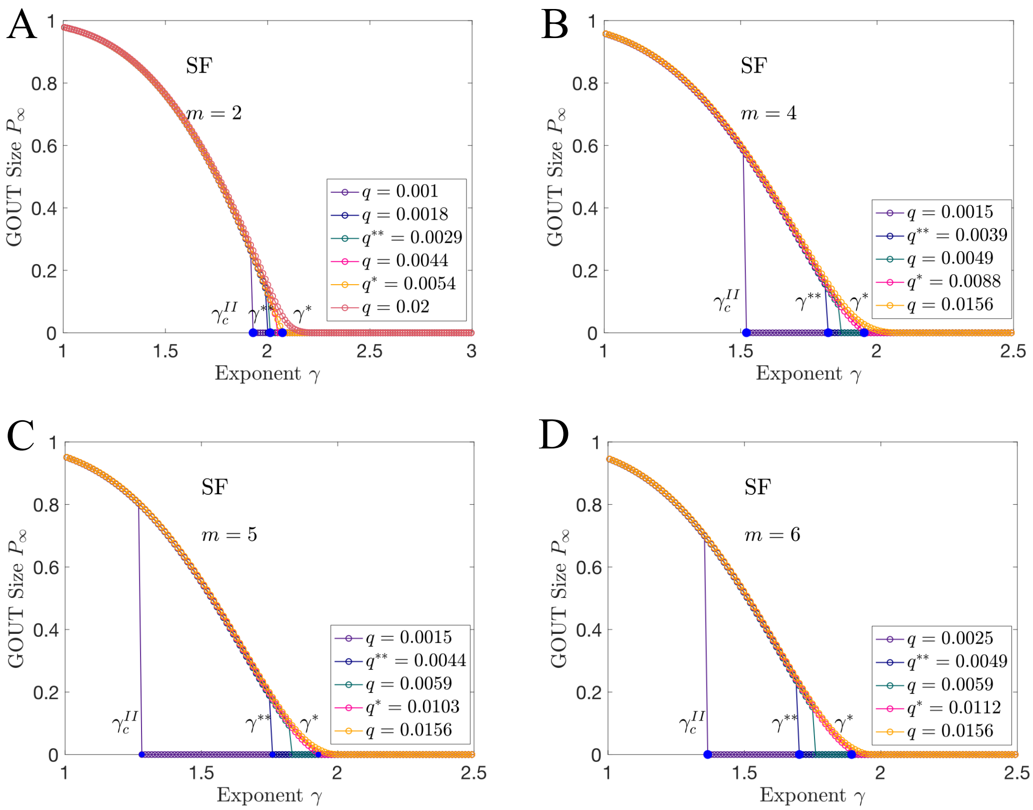

Fig. S11. The relation between GOUT size P_∞ and exponent γ in directed SF networks. There are first-order phase transitions, hybrid phase transitions and second-order(continuous) phase transitions when active node ratio $q < q^{**}$, $q^{**} < q < q^{*}$ and $q > q^{*}$. Curves and dots indicate theoretical solutions.

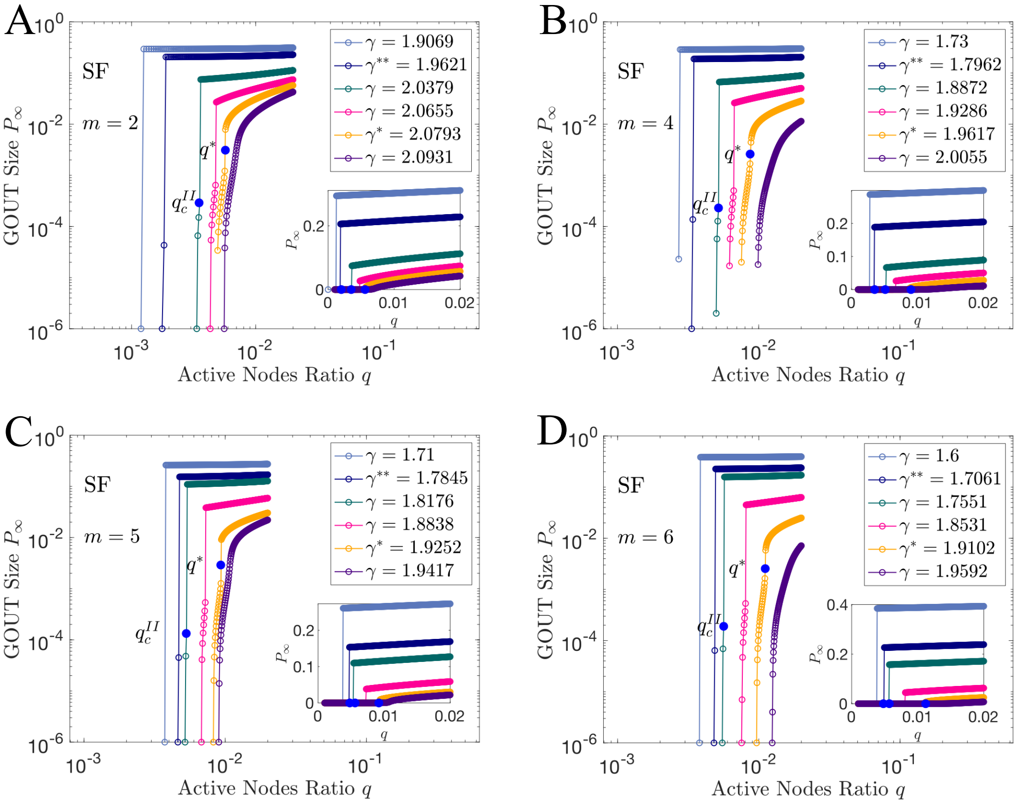

Fig. S12. The relation between GOUT size P_∞ and active nodes ratio q in directed SF networks. There are first-order phase transitions, hybrid phase transitions and second-order(continuous) phase transitions when active node ratio $\gamma < \gamma^{**}$, $\gamma^{**} < \gamma < \gamma^*$ and $\gamma > \gamma^*$. Curves and dots indicate theoretical solutions.

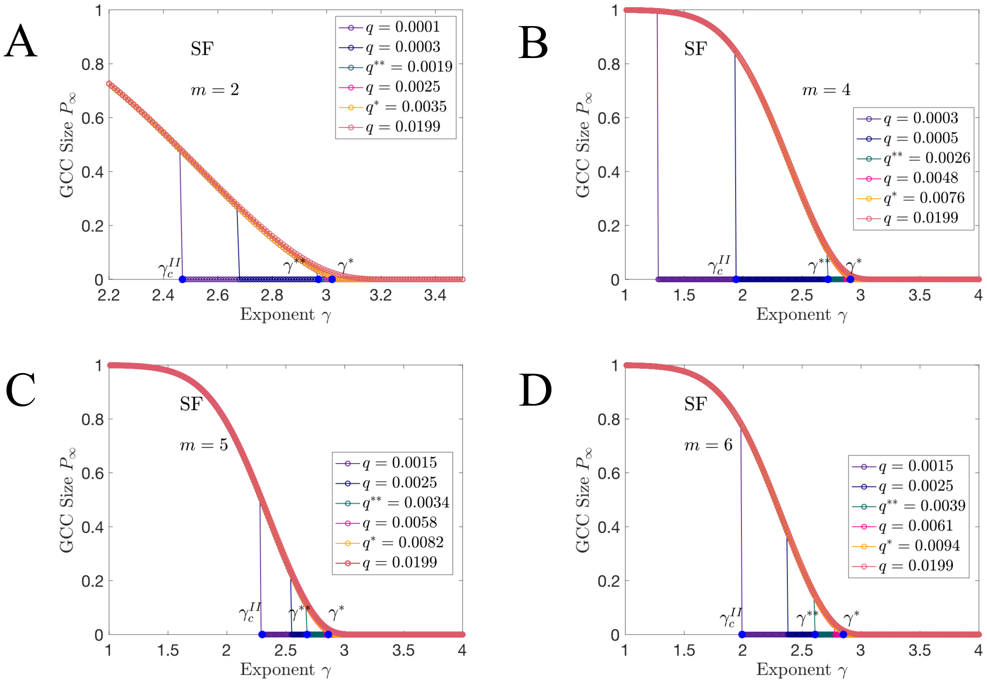

Fig. S13. The relation between GCC size P_∞ and exponent γ in undirected SF networks. There are first-order phase transitions, hybrid phase transitions and second-order(continuous) phase transitions when active node ratio $q < q^{**}$, $q^{**} < q < q^*$ and $q > q^*$. Curves and dots indicate theoretical solutions.

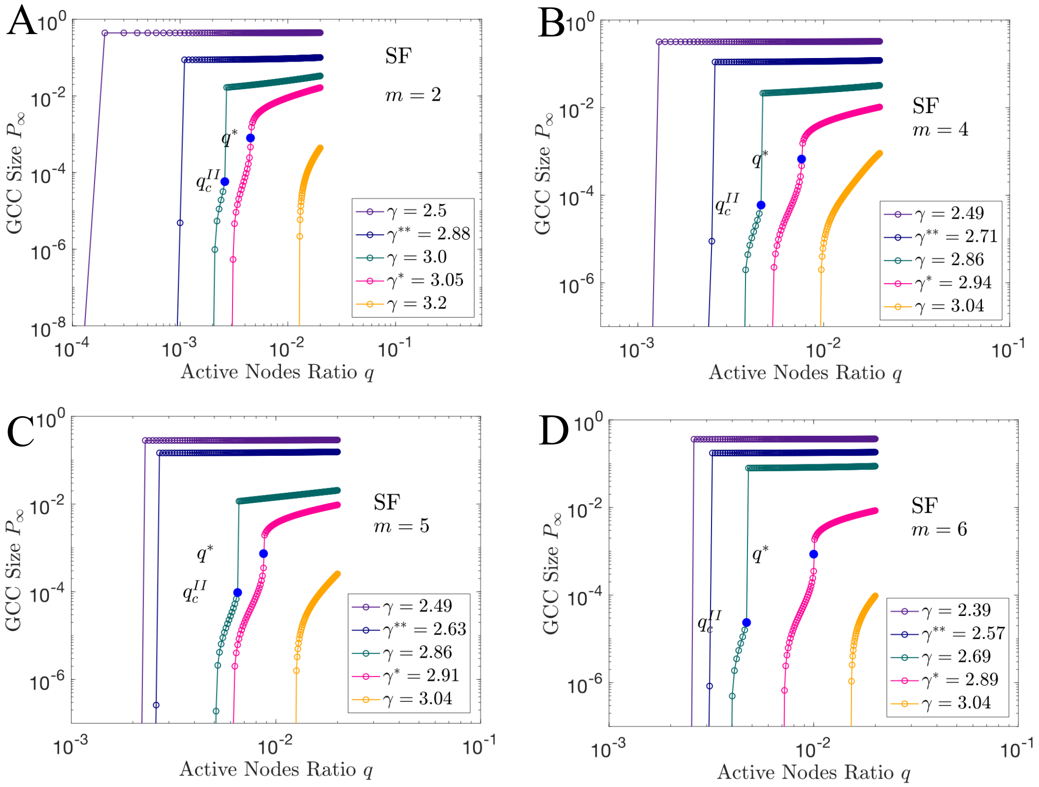

Fig. S14. The relation between GCC size P_∞ and active node ratio q in undirected SF networks. There are first-order phase transitions, hybrid phase transitions and second-order (continuous) phase transitions when active node ratio $\gamma < \gamma^{**}$, $\gamma^{**} < \gamma < \gamma^*$ and $\gamma > \gamma^*$. Curves and dots indicate theoretical solutions.

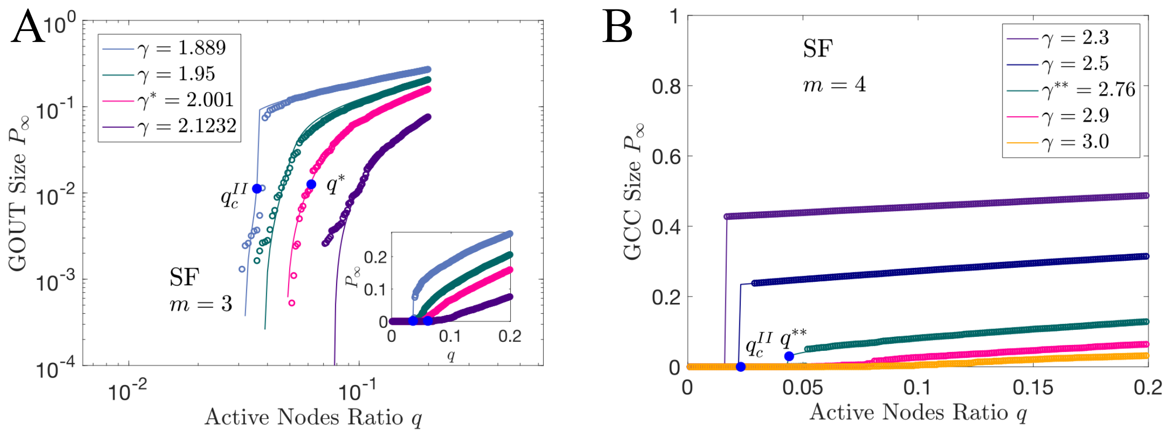

Fig. S15. Comparison between simulation and theoretical results in SF networks. **A** The relation between GOUT size P_∞ and active node ratio q in directed SF networks. There are hybrid and second-order(continuous) phase transitions. **B** The relation between GCC size P_∞ and active node ratio q in undirected SF networks. It is shown with hybrid and second-order(continuous) phase transitions. In all figures, curves represent theoretical results and dots represent simulation results in large networks with size 1,000,000.

Table S1. Descriptions of real-world networks.

Network	Type	Number of nodes	Number of edges
DBLP	Undirected	560,396	2,073,703
AstroPh	Undirected	18,772	198,110
CondMat	Undirected	23,133	93,497
Cite-HepPh	Directed	34,546	421,578
Cite-HepTh	Directed	27,770	352,807

Table S2. Definition of probabilities for the theoretical solution of growth-induced percolation in undirected networks. The first column is the probability corresponding to the event in the second column. $i-j-\infty$ denotes that i connects to the GCC through j for the randomly chosen link $i-j$. In the fourth column, s denotes the number of active neighbors of j (excluding i). In the fifth column, t denotes the number of neighbors of j (excluding i) that can induce j to state 1. The sixth column is the probability that both the event in the second column and the state of j in the third to the fifth columns are satisfied. $F(s)$ is the abbreviation of $F\left(\tilde{x}; k-1, s\right) = \binom{k-1}{s}\tilde{x}^s(1-\tilde{x})^{k-1-s}$. $G(t) = F\left(\frac{\tilde{y}}{\tilde{x}}; s, t\right) = \binom{s}{t}\left(\frac{\tilde{y}}{\tilde{x}}\right)^t\left(1-\frac{\tilde{y}}{\tilde{x}}\right)^{s-t}$.

Notation	Description of the event	The state of j			The probability of the event $\wedge$ the state of j
		Initial state	s	t	
$\tilde{x}$	The state of j is 1 without the induction from node i	1			q
		0			$(1-q)\left[1-(1-\tilde{y})^{k-1}\right]$
$\tilde{y}$	j can induce i	1	$s \geq m$		$qF(s)$
		0	$s \geq m$		$(1-q)F(s)\left[1-\left(1-\frac{\tilde{y}}{\tilde{x}}\right)^s\right]$
α	$i-j-\infty$ given i can induce j		$s \leq m-2$		$F(s)\left[1-\left(1-\frac{\beta}{\tilde{x}}\right)^s\right]$
			$s \geq m-1$		$F(s)\left[1-\left(1-\frac{\beta}{\tilde{x}}\right)^s\left(1-\frac{\alpha-\beta}{1-\tilde{x}}\right)^{k-1-s}\right]$
β	$i-j-\infty$, given the state of i is 1 but i can not induce j	1	$s \leq m-2$		$qF(s)\left[1-\left(1-\frac{\beta}{\tilde{x}}\right)^s\right]$
		1	$s \geq m-1$		$qF(s)\left[1-\left(1-\frac{\beta}{\tilde{x}}\right)^s\left(1-\frac{\alpha-\beta}{1-\tilde{x}}\right)^{k-1-s}\right]$
		0	$s \leq m-2$	$t > 0$	$(1-q)F(s)G(t)\left[1-\left(1-\frac{\gamma}{\tilde{y}}\right)^t\left(1-\frac{\beta-\gamma}{\tilde{x}-\tilde{y}}\right)^{s-t}\right]$
		0	$s \geq m-1$	$t > 0$	$(1-q)F(s)G(t)\left[1-\left(1-\frac{\gamma}{\tilde{y}}\right)^t\left(1-\frac{\beta-\gamma}{\tilde{x}-\tilde{y}}\right)^{s-t}\left(1-\frac{\alpha-\beta}{1-\tilde{x}}\right)^{k-1-s}\right]$
γ	$i-j-\infty$ without the effect of i on j	1	$s \geq m$		$qF(s)\left[1-\left(1-\frac{\beta}{\tilde{x}}\right)^s\left(1-\frac{\alpha-\beta}{1-\tilde{x}}\right)^{k-1-s}\right]$
		0	$s \geq m$	$t > 0$	$(1-q)F(s)G(t)\left[1-\left(1-\frac{\gamma}{\tilde{y}}\right)^t\left(1-\frac{\beta-\gamma}{\tilde{x}-\tilde{y}}\right)^{s-t}\left(1-\frac{\alpha-\beta}{1-\tilde{x}}\right)^{k-1-s}\right]$
